# Real-Time Monitoring of Breath Biomarkers with A Magnetoelastic Contactless Gas Sensor: A Proof of Concept

**DOI:** 10.3390/bios12100871

**Published:** 2022-10-13

**Authors:** Alvaro Peña, Juan Diego Aguilera, Daniel Matatagui, Patricia de la Presa, Carmen Horrillo, Antonio Hernando, Pilar Marín

**Affiliations:** 1 Instituto de Magnetismo Aplicado (IMA), Universidad Complutense de Madrid-Administrador de Infraestructuras Ferroviarias (UCM-ADIF), 28230 Las Rozas, Spain; 2 Departamento de Física de Materiales, Universidad Complutense de Madrid (UCM), 28040 Madrid, Spain; 3 Grupo de Tecnología de Sensores Avanzados (SENSAVAN), Instituto de Tecnologías Físicas y de la Información (ITEFI), Consejo Superior de Investigaciones Científicas (CSIC), 28006 Madrid, Spain; 4 Donostia International Physics Center, 20018 Donostia, Spain; 5 Instituto Madrileño de Estudios Avanzados (IMDEA) Nanociencia, 28049 Madrid, Spain; 6 Departamento de Ingeniería, Universidad de Nebrija, 28015 Madrid, Spain

**Keywords:** remote sensing, gas sensor, breath analysis, magnetoelastic resonance, soft magnets, polyvinylpyrrolidone, nanofiber, humidity, biomarkers, diabetes

## Abstract

In the quest for effective gas sensors for breath analysis, magnetoelastic resonance-based gas sensors (MEGSs) are remarkable candidates. Thanks to their intrinsic contactless operation, they can be used as non-invasive and portable devices. However, traditional monitoring techniques are bound to slow detection, which hinders their application to fast bio-related reactions. Here we present a method for real-time monitoring of the resonance frequency, with a proof of concept for real-time monitoring of gaseous biomarkers based on resonance frequency. This method was validated with a MEGS based on a Metglass 2826 MB microribbon with a polyvinylpyrrolidone (PVP) nanofiber electrospun functionalization. The device provided a low-noise (RMS = 1.7 Hz), fast (<2 min), and highly reproducible response to humidity (Δf = 46–182 Hz for 17–95% RH), ammonia (Δf = 112 Hz for 40 ppm), and acetone (Δf = 44 Hz for 40 ppm). These analytes are highly important in biomedical applications, particularly ammonia and acetone, which are biomarkers related to diseases such as diabetes. Furthermore, the capability of distinguishing between breath and regular air was demonstrated with real breath measurements. The sensor also exhibited strong resistance to benzene, a common gaseous interferent in breath analysis.

## 1. Introduction

Several gaseous pollutants, such as nitrogen dioxide, carbon monoxide, and volatile organic compounds (VOCs), are known to cause or exacerbate respiratory or other health-related problems [1,2,3,4]. Devices capable of quickly detecting these gases, even at low concentrations, are critical to minimizing exposure and, thus, preventing the associated hazards to human health [5,6].

In recent years, attention to technologies able to detect molecules in gaseous environments has awakened an enormous interest in the scientific community, especially in health-related applications [7,8,9,10]. Similarly, interest in technologies that allow development of low-cost devices with high sensitivity and low dimensionality able to detect molecules in gaseous environments has engendered substantial interest in the scientific community, especially in health-related applications. Among the most developed technologies, the solid-state chemical sensors stand out, which are based on different physical magnitudes as impedance, resistivity, and piezoelectricity [1,2,3,4], as well as those based on acoustic waves [11,12] where the detection process is based on the changes caused by acoustic waves propagating through a piezoelectric substrate where the frequency variation makes it possible to quantify the concentration of gas in the sensor environment. They show high sensitivity, fast response, low cost, and work at room temperature, but require a complex instrumentation (electrical wiring), which limits application possibilities.

The challenge is to establish new analytical systems with sufficient ability and portability as an alternative to the conventional analytical systems [13], which are accurate, but bulky, expensive, and require highly qualified operators. These facts lead to the conventional systems not being used for applications where an analytical study is required, such as air quality, safety, or medicine [14]. Among medical applications, sensing of gaseous biomarkers, molecules found in exhaled human breath which are related to abnormal biological processes or exogenous factors, is a handy tool for early diagnosis and monitoring of diseases [15,16]. Furthermore, presence of these molecules may be affected by factors such as smoking, drugs, age, diet, or body mass index [17,18,19].

For instance, ammonia generates great interest as a biomarker [20]. Abnormal ammonia concentrations in the exhaled breath have been mainly related to type 2 diabetes [21] and bacterial infection [22], kidney malfunction [23], or asthma [24].

Acetone is another important biomarker related to blood glucose concentration in diabetic patients [25,26]. Therefore, ammonia and acetone detection as gaseous biomarkers, as opposed to blood glucose meters that require punctures, could represent a non-invasive, convenient, and harmless alternative in the day-to-day monitoring of diabetic patients [21,27].

Other biomarkers may originate from exogenous factors, such as benzene. For example, significant benzene concentrations have been detected in breath as a result of smoking habits or air pollution. In such cases, this biomarker manifests the benzene accumulated in the body [18,19]. However, the particular interest in benzene stems from the fact that it can act as an interferent and disturb the operation of gas sensors targeting different biomarkers.

Another interesting analyte for gas sensors is water in the gas phase. The body naturally generates it in high concentrations; therefore, humidity sensors have become a powerful tool in biomedical applications. For example, real-time humidity monitoring allows discrimination between exhaled and regular ambient air; therefore, these biosensors would help detect irregular breathing due to sleep apnea, asthma, or cardiac arrest [10,28].

As it is well known, magnetoelastic devices greatly appeal to sensor development in biological and medical applications owing to their intrinsic wireless operation properties [29,30]. They are usually made from amorphous ferromagnetic ribbons [31] or wires [32], primarily iron-rich alloys with a combination of high mechanical strength (~1000–1700 MPa), a high magnetoelastic coupling coefficient (k), up to 0.98, and magnetostriction on the order of 10^−5^ (dimensionless) [33,34]. For magnetoelastic microribbons, the highest k values have been obtained by thermal annealing under a magnetic field perpendicular to the ribbon’s axis that induces a transverse homogeneous easy axis [35]. The high magnetoelastic coupling, which depends upon the ease of rotation of magnetization, allows an efficient conversion from magnetic to elastic energy and vice versa. The magnetization variation leads to the generation of mechanical stresses and deformations (Villari or magnetostrictive effect [36]) and, at the same time, the application of mechanical stresses can lead to changes in its magnetic properties, mainly related to the anisotropy and susceptibility (Joule or magnetoelastic effect [37]). 

Under an alternating magnetic field, a periodic shape modification, i.e., a vibration, can be achieved. When the stationary mechanical wave matches the length of the propagation axis in the solid (such as L = n (λ/2), with n being an integer other than zero), the magnetoelastic resonance frequency appears [38]. This resonance can be extremely sensitive to several external parameters and, consequently, the transducer can act as a standalone sensor for monitoring temperature and magnetic fields [39,40], density and viscosity of a medium [41,42,43], or mechanical stresses, including tension, torsion, or pressure [33,44,45].

However, the most exciting applications for these transducers are achieved upon functionalization with sensitive layers. These layers typically consist of a material that responds to external parameters by changing its mass. As the mass of the active layer changes with an external parameter, the resonant frequency of the transducer also varies. This principle has been used to develop magnetoelastic sensors (MESs) for viruses [46], bacteria [47,48,49], biomolecules [50,51], biological processes [52,53], or gaseous molecules [54,55,56].

Magnetoelastic resonance-based gas sensors (MEGSs) are particularly appealing for breath analysis applications, as previously discussed, owing to their low cost, remote operation, and unneeded integrated power source, as they only operate under the externally applied magnetic field [38,57]. In addition, this enormous advantage means they can be easily miniaturized or made into portable sensors [58,59].

Effective diagnosis requires detecting a mixture of biomarkers, called exhaled breath profiles, which can contain more than 3000 compounds [60]. This task often requires devices with an array of sensors with different selectivity. However, such complexity falls out of the scope of the present work, which mainly focuses on the potential of MEGSs for developing real-time monitoring of exhaled breath biomarkers.

Typical MEGSs operation relies on a magnetic field frequency sweep to find the resonance frequency [54,55,56,61]. Although this technique reveals several magnetoelastic parameters, it slows down the resonant frequency monitoring (steps of tens of seconds at best), making it unable to monitor fast reactions in real time and, consequently, impeding its use in practical breath analysis applications.

This work presents a proof of concept of the development of a magnetoelastic transducer and a measuring setup able to characterize resonant frequency in real time. This transducer was functionalized with nanofibers of a sensitive polymer, polyvinylpyrrolidone (PVP), to build a sensor capable of distinguishing between regular air and exhaled breath, as well as of quantitative and reproducible detection of relative humidity (RH), acetone, and ammonia in gaseous environments in a contactless, remote manner. In addition, benzene was used to test the sensor selectivity capacity.

## 2. Materials and Methods

### 2.1. Magnetoelastic Sensor Device

The transducer used for this work was a 37.00 × 6.60 × 0.02 mm magnetoelastic microribbon (2826MB, Metglas, Conway, SC, USA).

The sensitive layer consisted of electrospun PVP nanofibers directly deposited over the microribbon with an electrospinning method. First, PVP (Mw = 360,000 g/mol, ref: 81440 Sigma-Aldrich, Burlington, MA, USA) was dissolved in distilled water at a 1:4 weight ratio and stirred until homogeneous. The solution was then degasified under vacuum conditions until any visible air bubbles were removed. Next, the solution was loaded into a syringe with a metal needle connected to a high-voltage power supply and placed at a 14 cm distance from the microribbon that acted as the grounded collector. Finally, the solution was extruded at a flow rate of 5 μL/min for 30 min at a voltage of 14 kV. The electrospinning parameters were chosen according to an optimum configuration [62].

The morphology and distribution of the PVP nanofibers were studied with scanning electron microscopy (JEOL JSM 6335F, Centro Nacional de Microscopía Electrónica, Madrid Spain).

### 2.2. Sensor Cell

**Figure 1:**
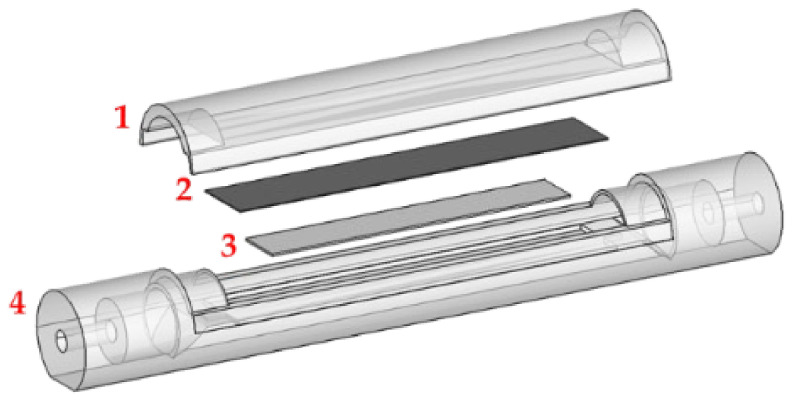
Schematics of the sensor cell, which includes cover (1), magnetoelastic microribbon (2), permanent magnet (3), and main body (4).

The MEGS based on the functionalized transducer was placed, with the deposited side up, in a custom-made 3D-printed PLA airtight cell (with a volume of ~15 mL) connected to an automatized gas sample generator. Inside the cell was a 30.85 × 6.00 × 0.05 mm permanent magnet (~517 A/m) (Figure 1). The magnet creates a bias field near the ribbon’s anisotropy field, which induces magnetization in the transducer, typically between 200 and 900 A/m [63]. This magnetization maximizes the magnetoelastic coupling coefficient value and improves magnetoelastic effect detection [35,64] (Figure 1).

### 2.3. Magnetoelastic Resonance Oscillator Circuit

**Figure 2:**
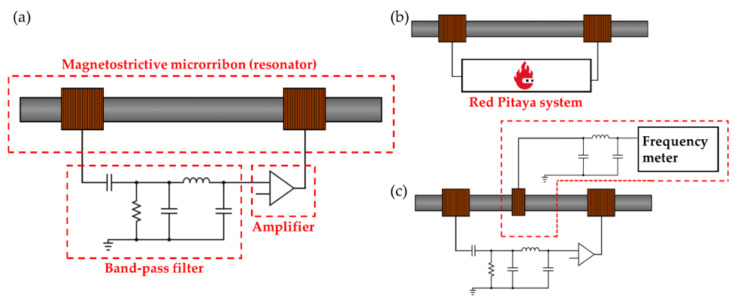
(**a**) Schematics of the oscillator circuit and electrical characterization setups for (**b**) frequency spectra and (**c**) real-time oscillator monitoring.

An oscillator circuit was designed around the sensor cell to allow real-time monitoring of the resonance frequency (Figure 2a). Due to the characteristic change in the susceptibility of the magnetoelastic transducer, the circuit will oscillate, with a radiofrequency (RF) signal travelling the loop that matches the transducer’s resonance frequency [65]. The oscillator system comprised of three basic circuit blocks:

- An amplifier;
- A feedback network (magnetostrictive resonator);
- A passive bandpass filter.

The amplifier was connected to a power source, and was the only active element of the loop. Amplifier noise induces random multiple-frequency signals in the loop, from which the resonance frequency is the only one allowed to travel through the entire loop. The Barkhausen criteria should be satisfied, with a total phase shift of 2πn (with n being an integer other than zero) and a loop gain equal to the unity to ensure that the system works as an oscillator. When these criteria are fulfilled, the frequency of the oscillator circuit is autonomously and immediately synchronized upon any disturbance in the transducer.

The magnetostrictive oscillator included the sensor cell and a set of twin coils, with a diameter of 12 mm, a length of 17 mm, and 300 turns of 0.15 mm copper wire. These coils were coupled on the cell’s sides and acted as input and output RF ports. This configuration of lateral coils, instead of the traditional concentrical disposition [40], was chosen so that the prototype could exploit the contactless nature of the device operation.

Finally, the passive band filter selected the second resonance frequency harmonic and excluded interferent signals from external sources.

### 2.4. Electrical Characterization and Data Acquisition

#### 2.4.1. Magnetoelastic Resonance Analysis

For the magnetoelastic characterization, a Red Pitaya device (SDRlab 125-14, Red Pitaya, Solkan, Slovenia) was controlled through a custom-made LabView-based program, as used in a previous report [40] (Figure 2b). Briefly, the Red-Pitaya-based system generates an alternating magnetic field through any of the twin coils previously described that, in this configuration, acts as an exciting coil while simultaneously collecting the signal induced in the other coil, working as a pick-up coil in this configuration (Figure 2b). The system sweeps the frequency over a predefined range and provides the frequency spectra (amplitude (dB) vs. frequency (Hz)).

#### 2.4.2. *Q* Factor Determination

The *Q* factor, also called the quality factor, is the parameter that measures the ratio between the energy stored and the energy dissipated during a complete signal cycle. A high factor indicates a low rate of energy loss relative to the energy stored by the resonator (Equation (1)) [66,67]:

(1) $$Q\overset{\mathrm{def}}{=}2\pi \times \frac{energy\;stored}{energy\;dissipated\;per\;cycle}$$

The quality factor can be estimated experimentally as Equation (2):

(2) $$Q=\frac{f_{c}}{BW}$$

where $f_{c}$ is the peak center and BW is the 3 dB bandwidth (from the maximum amplitude). A high *Q* factor value indicates a sharp resonance peak and, therefore, good resolution when determining frequency shifts [67].

Under continuous-wave operation, the amplifier can compensate for the losses of the magnetoelastic resonator in the oscillator. When such gain is considered, the *Q* factor relates to the whole oscillator system instead of the magnetoelastic resonator alone. For the determination of the effective *Q* factor, high-resolution measurements of the line width of the frequency spectrum were obtained with a spectrum analyzer (9320A, Agilent, Santa Clara, CA, USA). The configuration for these measurements was similar to the one shown in Figure 2c, substituting the frequency meter with the spectrum analyzer.

For the real-time monitoring during the sensor operation, the signal travelling through the oscillator system was detected using a pick-up coil, with a diameter of 12 mm, length of 2 mm, and 35 turns of 0.15 mm copper wire, connected to a frequency meter (53131A, Agilent, Santa Clara, CA, USA). The pick-up coil included a low-pass filter to block the 50 Hz range (Figure 2c). Note that the pick-up coil samples the alternating field travelling through the loop emitted by the coils. As such, it could be placed in a different configuration. 

Finally, to illustrate the effect of the magnetoelastic resonance on the oscillator system, computational simulations were carried out over the magnetoelastic microribbon and the twin coils with COMSOL Multiphysics (version 5.5, AC/DC module, Barcelona, Spain). Susceptibility was parametrized with a relatively high value for the resonance case and a relatively low value for the non-resonance case, both values being arbitrary.

### 2.5. Sensors Evaluation

The sensor was tested with sequences consisting of a baseline step, where only synthetic dry air was flushed in the sensor chamber, cycles with an exposure phase, where the sensor was tested with the gas mixture containing the analyte, and a purge phase, where the device was again exposed only to the carrier gas.

For the humidity test, synthetic air was passed through a water bubbler and mixed with the carrier to create different humidity conditions to which to expose the device. The mix was calibrated with a humidity and temperature meter (RS 1364, RS Components, London, UK) to achieve the desired RH values. The sequence consisted of a 60 min baseline step followed by three cycles at each RH level (17%, 36%, 54%, 73%, and 95%). Each cycle had a 5 min exposure and a 10 min purge phase.

For the acetone, ammonia, and benzene tests, the analytes (from 50 ppm balance air cylinders) were mixed, and synthetic dry air used as a carrier at a concentration of 40 ppm. The sequences consisted of three different runs, one for each analyte, with a 60 min baseline step and three cycles, with a 2 min exposure phase and a 5 min purge phase.

**Figure 3:**
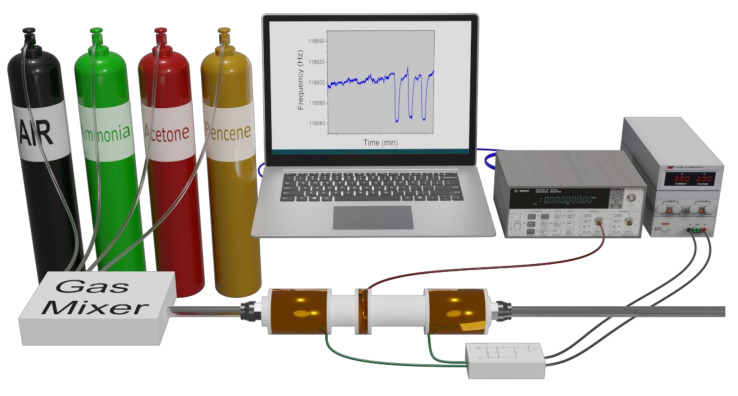
Experimental setup for the real-time monitoring of the magnetoelastic resonance-based gas sensor.

The experiments were performed in a temperature-controlled room at 25 °C. The airflow inside the cell was set to 100 mL∙min^−1^ in all cases. All gases were provided by Nippon Gases, Madrid, Spain. The complete experimental setup is illustrated in Figure 3.

Three parameters were used to evaluate the sensor’s performance: response, limit of detection (*LoD*), and τ90.

The sensor’s response was defined as the frequency shift value (Δ*f*) between the exposure phase beginning (*f_i_*) and end (*f_f_*) (Equation (3)):

(3) $$∆f=f_{r}-f_{i}$$

*LoD* represents the minimum concentration of the analyte that can be reliably detected. It can be calculated from the sensitivity using the following Equation (4):

(4) $$LoD_{\left(RMSnoise,\;\mathrm{Sensitivity}\right)}=\frac{3\;*\;RMSnoise}{Sensitivity}$$

The RMS noise is the root mean squared noise of a 60 min baseline, i.e., under the continuous flush of the carrier gas in the chamber with no analyte present. The *LoD* error (Δ*LoD*) was calculated from Equation (4) using uncertainty propagation.

Finally, τ90 was used to evaluate the responsiveness time. It was defined as the time needed to achieve the 90% of the response.

### 2.6. In-Situ Breath Detection

The breath monitoring capability of the device was evaluated by exposing the sensor to the breath of a researcher who breathed normally at 5 cm from the sensor.

The experiment was carried out with the same configuration used for *Q* factor determination, i.e., with the spectrum analyzer and the cover of the gas chamber removed. This configuration was used, instead of the oscillator system, to detect any damping on the oscillation amplitude or signal distortion.

## 3. Results and Discussion

### 3.1. Sensitive Layer Deposit

**Figure 4:**
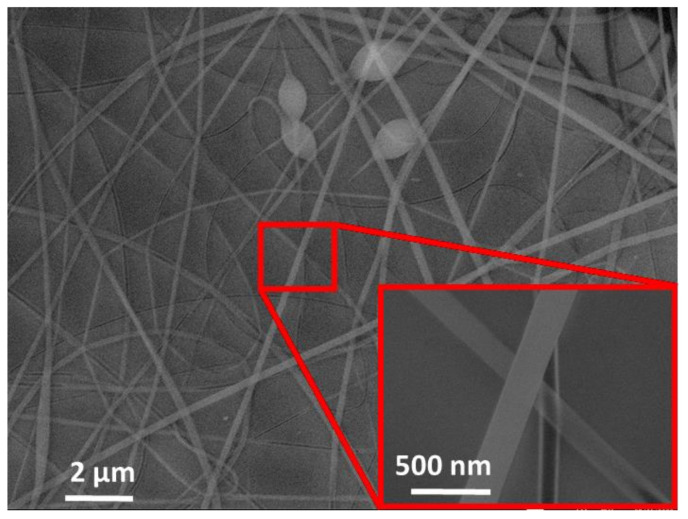
SEM images of the electrospun sensitive layer deposited over the transducer.

The sensitive layer was successfully deposited over the transducer. Due to the characteristics of the electrospinning technique, the water-based PVP solution dried and formed polymeric nanofibers, with a thickness in the 100 nm range, on the target (Figure 4).

The nanofibers were evenly deposited on the transducer’s surface mainly due to the good electrical conductivity of the magnetoelastic microribbon, which promoted a homogeneous electric field distribution during the electrospinning process.

### 3.2. Magnetoelastic Resonance Characterization

**Figure 5:**
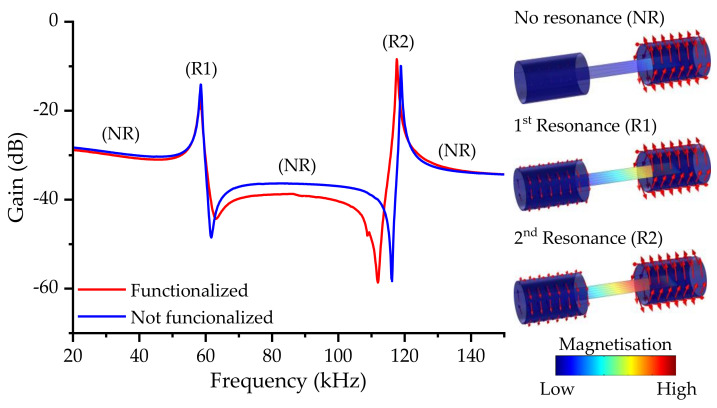
Frequency spectra of the transducer with and without the sensitive layer functionalization. Simulation results are used to illustrate the magnetoelastic resonance in the gain spectra for the no resonance state (NR) and the first (R1) and second (R2) harmonic of the magnetoelastic resonance.

During the frequency sweep, as the magnetoelastic resonance is achieved, the change of the magnetic susceptibility leads to an increase in its magnetization and, consequently, to a higher induced current, or gain, in the pick-up coil [66]. Thus, the magnetoelastic resonance appeared as amplitude maxima in the frequency sweep spectra (Figure 5). 

In general, the first harmonic is usually greater when centered coils are used, but, in this case, due to the placement of the coils on the magnetoelastic ribbon sides, the second harmonic was favored against the first.

**Figure 6:**
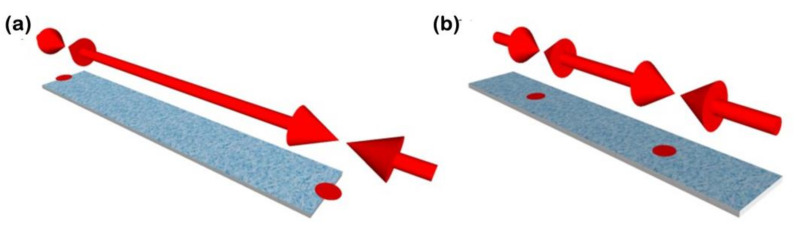
Schematic representation of the hot sensing areas for (**a**) the first and (**b**) second harmonics of the magnetoelastic resonance.

Furthermore, as the different harmonics have a different displacement distribution in terms of the oscillation, they also have areas with higher sensitivity, often called hot sensing areas, which, for the first harmonic, are located in the tips of the ribbon [38] (see Figure 6).

For the reference transducer, i.e., without functionalization, the first harmonic was located at 58.6 kHz, whereas the second harmonic was located at 118.8 kHz, roughly twice the value. These values agree with the ones calculated from the mechanical parameters of the magnetoelastic microribbon (Equation (5)) [57]:

(5) $$f_{r}=\frac{n}{2L}\sqrt{\frac{E}{\rho \left(1-\upsilon^{2}\right)}}$$

Considering the length of the microribbon and its mechanical properties (*E* = 152 GPa; density = 7900 kg/m^3^; Poisson ratio = 0.33) [68], the theoretical $f_{r}$ values were 62.8 kHz and 125.6 kHz for *n* = 1 and *n* = 2, respectively. Differences between the theoretical and experimental values may be explained by the fact that Equation (5) does not consider the magnetic variation of the mechanical parameters [69], nor does it consider deviations in the materials parameters, because of the manufacturing process.

As the functionalization process adds mass to the transducer, a shift in the resonance frequency is expected according to Equation (6):

(6) $$∆f=-f_{0}\frac{∆m}{2m_{0}}$$

where $m_{0}$ and $f_{0}$ are the mass and resonant frequency of the magnetoelastic material, respectively, prior to the coating [57]. It should be noted that, although Equation (2) assumes that the coating has rigidly adhered to the substrate, it is uniformly distributed on the transducer surface, and the deposited mass is relatively small compared to $m_{0}$, which may not always be satisfied [70]. Nevertheless, the general assumption that an increment in the sensor’s mass leads to a negative frequency shift is commonly accepted. This shift was confirmed with the frequency sweep spectra of a transducer with and without the functionalization.

At the same time, the deposit had a neglectable effect on the dampening of the resonance amplitude, which remained largely unaltered, indicating the robustness of the magnetoelastic strips as a transducer for MEGSs. 

The second harmonic of $f_{r}$ was chosen for the MEGS operation because of its higher amplitude, which should also improve the sensing performance, and its intrinsic higher sensitivity (higher $f_{0}$, Equation (6)).

**Figure 7:**
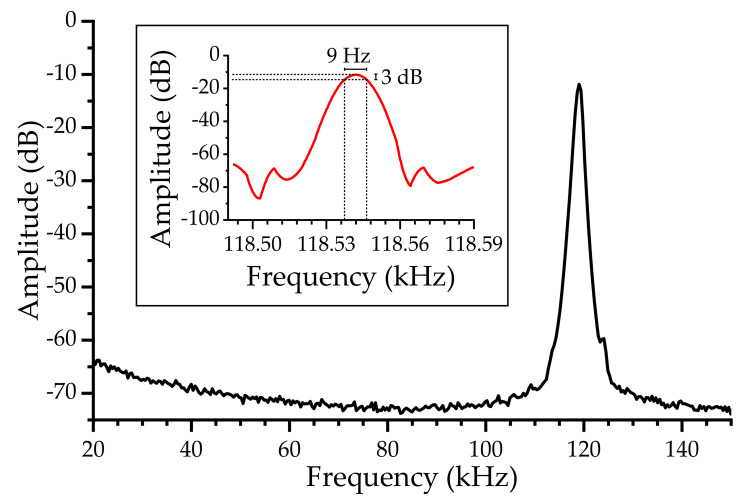
High-resolution frequency spectra for the quality factor determination with 13 Hz steps (black line) and 0.2 Hz steps (red line).

The oscillator’s quality factor was calculated using Equation (2) and a high-resolution measure (Figure 7). With the linewidth of 9 Hz for a frequency of ~119 kHz, a high-quality factor of Q_oscillator_ > 13,000 was obtained.

### 3.3. Gas Response Characterization

#### 3.3.1. Humidity and Real Breath Monitoring

**Figure 8:**
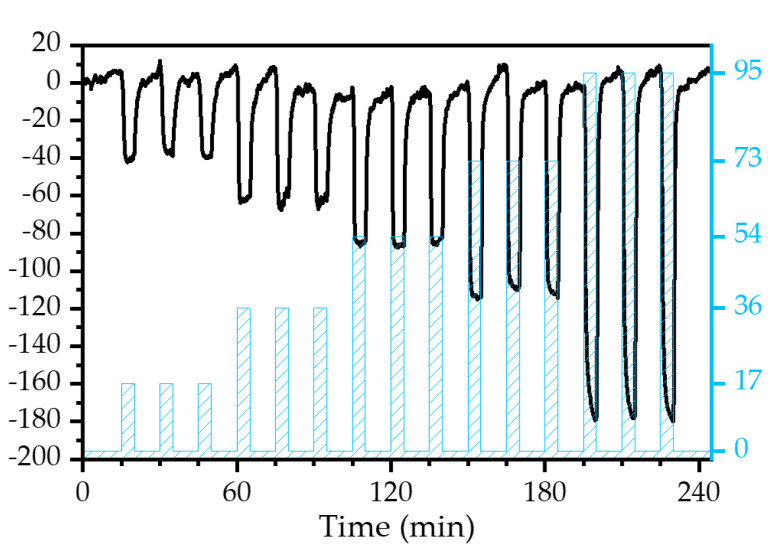
Real-time frequency shift of the device exposed to different RH levels.

The viability of the experimental setup, including the sensor, was first evaluated toward gaseous water molecules detection, i.e., relative humidity measurement. As water acts as a solvent of PVP, H_2_O molecules are trapped in the sensitive material’s nanofibers, incrementing the deposited mass over the transducer. A negative resonant frequency shift, as predicted by Equation (6), was observed during the real-time monitoring under the exposure to different RH levels (Figure 8).

**Figure 9:**
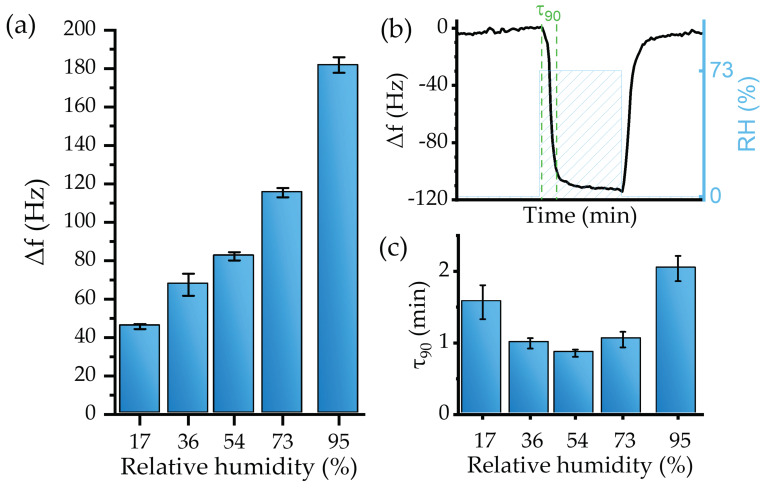
(**a**) Calibration curve, (**b**) τ_90_ calculation method, and (**c**) τ_90_ values for the sensor humidity test.

The sensor responded with high reproducibility. For instance, after three exposures to the same RH level, the relative standard deviation of the response was ≤4% (Figure 9a).

The sensitivity of the device, defined as the response to a specific concentration of the analyte (expressed in Hz/%), was obtained by fitting the values provided in Figure 9a. A linear response regime for levels up to RH = 73% was established. The linear fit provided a sensitivity of −1.17 ± 0.1 Hz/% (R^2^ of 0.97). 

However, for high RH, i.e., 95%, the sensors’ behavior deviated from the linearity. This effect may be explained by a large total mass of the active layer relative to the mass of the transducer [70] or, most probably, by condensation effects. Mean values and standard deviation of the sensor’s response toward RH are presented in Figure 9a. The resulting LoD, calculated from Equation (4), was an RH level of 4.7 ± 0.4%.

**Figure 10:**
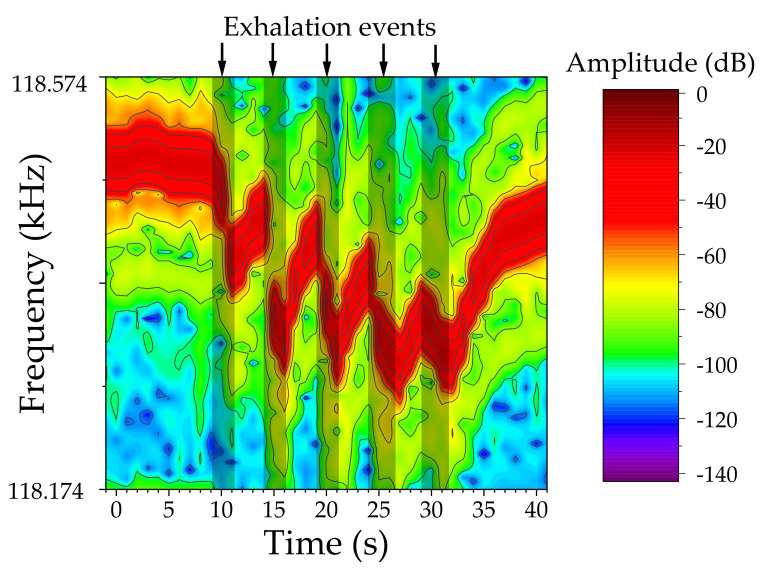
Breath detection test, the resonance frequency (red area) shifts to lower values as a response to exhalation events.

The responsiveness time can be evaluated using the τ_90_ parameter, defined as the time required to achieve the 90% maximum frequency change (Figure 9b). In general, the sensor device showed a fast recovery with a low baseline drift for tested RH. For instance, Figure 10b shows sensor dynamics at 73% RH, where the response time was 1.04 min, which is similar to 1.04 min recovery time. Figure 9c shows that the sensor’s responsiveness toward water is fast, as 90% of the maximum change was achieved during the first two min or less. 

Although the resulting τ_90_ indicated fast responsiveness, mixing the analyte and the effect of the water bubbler could significantly delay the device’s response, as was also observed during the calibration with the hygrometer. 

The sensor was then exposed to natural breath; during this experiment, a researcher breathed normally onto the sensor. As the exhaled breath reached the device, an immediate response was observed. The response, consisting of a negative frequency shift, was most probably caused by a broad set of analytes in the breath, but mainly by its high humidity concentration (Figure 10). Although the exact compositional profile cannot be obtained with the current device, the excellent response to natural breath proved the utility of the sensor in breath analysis applications. Furthermore, the resonance amplitude remains largely unaltered, indicating that the transducer’s resonance did not suffer any noticeable damping or distortion during the breath exposures. 

#### 3.3.2. Endogenous Biomarkers: Acetone and Ammonia

**Figure 11:**
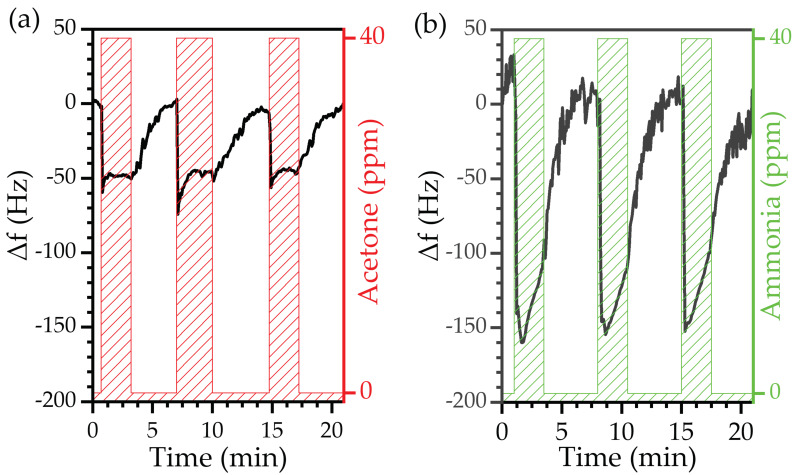
Real-time frequency shift of the device exposed to 40 ppm of (**a**) acetone (gas) and (**b**) ammonia (gas).

The MEGS was then evaluated for acetone detection. The sensor responded to 40 ppm of gaseous acetone with a negative frequency shift, analogously to the behavior observed during the humidity detection test related to the absorption of acetone molecules in the sensitive layer (Figure 11a). Acetone detection exhibited high reproducibility with slight variation between exposure cycles. The mean response value was 44.1 ± 4.8 kHz.

The test was repeated for ammonia detection under the same conditions. The sensor’s response also indicated that the ammonia molecules became attached to the sensitive layer, as observed during the humidity or acetone tests (Figure 11b). The device exhibited a similar response among the three exposures, further confirming the excellent reproducibility of the MEGSs operation. The response was higher than that to the acetone exposure, with a mean value of 112.6 ± 11.0 kHz. The sensor showed an immediate response toward acetone and ammonia. However, a higher time was required to recover the base frequency; this is related to a slowing down of the diffusion of acetone and ammonia molecules within the PVP nanofibers with respect to water molecules.

Overall, the MEGS detected acetone and ammonia at a 40 ppm concentration. Furthermore, the sensor exhibited high reproducibility, mainly owing to the practically full recovery during the purge phase, i.e., the frequency achieved its initial value (frequency shift equal to zero), a phenomenon related to good desorption dynamics.

#### 3.3.3. Exogeneous Biomarkers: Benzene

**Figure 12:**
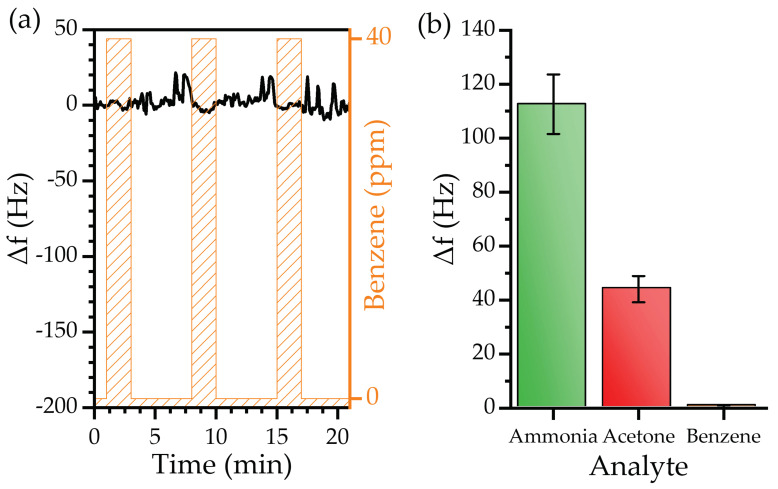
(**a**) Real-time frequency shift of the device exposed to 40 ppm of benzene (gas) and (**b**) comparison between the MEGS response to 40 ppm of ammonia, acetone, and benzene.

The last test was performed to study the effect of a common interferent biomarker in breath analysis, benzene (Figure 12a).

The MEGSs exhibited a neglectable response to 40 ppm of benzene, with a mean value of 0.5 ± 0.5 kHz. Compared to water, acetone, and ammonia, the sensor demonstrated an excellent performance, as the device showed an insignificant response to benzene. According to the relationship between the experimental responses, PVP nanofibers can bind with polar molecules, e.g., water, acetone, or ammonia with insignificant interaction with non-polar molecules such as benzene. In addition, the final selectivity is the product of many determinants of other solvation parameters [12,71]. The comparison between the three tested biomarkers highlights the fact that the device can target acetone and ammonia biomarkers without interference from benzene presence in exhaled breath (Figure 12b).

Table 1 presents a bibliographic compilation of gas sensors based on different technologies and compares them to our results. As it can be seen, the solution presented in this work and the high selectivity of the sensors open a new and promising field of research for magnetoelastic gas sensors, allowing the use of sensor arrays to classify diseases through exhaled breath.

## 4. Conclusions

In this work, we presented the development of a magnetoelastic resonance-based sensor with outstanding features like its real-time monitoring, contactless operation, low cost, and easy adaptability for monitoring of various gas biomarkers in exhaled breath. This proof of concept was based on three main components: a measuring system, a magnetoelastic ribbon transducer, and a sensitive layer.

The measuring system was designed for real-time monitoring of the resonance frequency and represents a significant breakthrough for the application of magnetoelastic resonance-based devices. 

The sensitive layer consisted of PVP nanofibers electrospun on the magnetoelastic ribbon. With this functionalization, the device exhibited a good response toward humidity, acetone, and ammonia, with no effects from interferences like benzene.

The device was capable of relative humidity monitoring in a fast (<2 min) and reproducible manner. With a sensitivity linear up to 73% and a slope of −1.17% (0.1 Hz/%) added to a low RMS noise, the sensor’s limit of detection was as low as (5% RH). Moreover, the sensor showed an excellent response when exposed to human breath.

The sensor was able to detect up to 40 ppm of acetone or ammonia well within the exposure time (2 min), with excellent recovery and reproducibility. Future work will focus on improving the device toward sub-ppm detection by introducing modifications in the sensitive layer or in the transducer’s geometry.

It is worth noting that the device’s response was monitored in real time with swift data acquisition compared to traditional magnetoelastic sensors. This real-time monitoring sensor can pave the way for a new kind of sensor of gas biomarkers for medical applications.

## Figures and Tables

**Table 1:** Comparison table for different sensor technologies examples.

Techn.	Main approach	Wireless	Analyte	Measurement	Response/Recovery Time	Ref.
CR	Silicon nanowires array	No	NH_3_/Air–100 ppb	Real time	~20 s both	[72]
CR	Tin dioxide @polyaniline composite	No	NH_3_/Air–100 ppm	Real time	125 s/167 s	[73]
CR	Cobalt phtalocyanines and reduced graphene oxide nanohybrids	No	NH_3_/Air–100 ppb	Real time	Response time 45 s, UV light used to speed up recovery	[74]
CR	SnO_2_ nanosheet	No	Acetone/Air–1 ppm	Real time	40 s/610 s	[75]
CR	Ru-doped SnO_2_ nanofibers	No	Acetone/Air–100 ppm	Real time	1 s/36 s	[76]
CR	Hollow Quasi-Graphite capsules/Polyaniline hybrid	No	NH_3_/Air–100 ppm	Real time	34 s/42 s	[77]
Opt.	Near Field Communication (NFC) technology, white LED as excitation source	Yes	NH_3_/N_2_–0.15% vol	Real time	5.43 min/6.64 min	[78]
Cap.	HF RFID (High-Frequency Radiofrequency Identification) smart label	Yes	NH_3_/Air–5 ppm	30 s each measure, 1 min period	~30 min	[79]
CR			RH–20 to 40%		5.43 min/6.64 min	
CR	Oxygen plasma-treated carbon nanotubes	Yes	NH_3_/Air–0–5 ppm	30 s each measure, 1 min period	~30 min	[80]
ME	TiO_2_ nanotubes on amorphous ribbon	Yes	RH–20 to 40%	4 s between each measurement	5.43 min/6.64 min	[81]
ME	BAYHYDROL-110 on Metglas ribbon	Yes	RH–10 to 80%	1 min between each measurement	Few min	[82]
ME	Poly (acrylic acid co-isooctylacrylate) on Metglas ribbon	Yes	NH_3_/N_2_–0.8% vol	1 min between each measurement	~15 min	[55]
			RH–0 to 15%		~1 min/several min	
ME	PVP nanofiber-functionalized micro ribbon	Yes	RH–17 to 95%	Real time	<2 min	This work
			Acetone/Air–40 ppm			
			NH_3_/Air–40 ppm			
			Benzene/Air–40 ppm			

Abbreviations: CR: Chemoresistive sensors; Opt.: Optical; Cap.: Capacitive; ME: Magnetoelastic resonance-based sensors.

## Data Availability

Not applicable.
